# (2-Methyl-4-oxo-4*H*-pyran-3-olato-κ^2^ *O* ^3^,*O* ^4^)bis­(triphenyl­phosphane-κ*P*)copper(I)–triphenyl­phosphane–methanol (1/1/1)

**DOI:** 10.1107/S1600536811011470

**Published:** 2011-04-13

**Authors:** Fabian M. A. Muller, Theunis J. Muller, Gideon Steyl

**Affiliations:** a Department of Chemistry, University of the Free State, PO Box 339, Bloemfontein 9300, South Africa

## Abstract

In the title compound, [Cu(C_6_H_5_O_3_)(C_18_H_15_P)_2_]·C_18_H_15_P·CH_3_OH, the pyran-4-one ring is appromimately planar (r.m.s deviation = 0.0138 Å), with the Cu^I^ atom 0.451 (5) Å out of the plane. The Cu^I^ atom has a distorted tetra­hedral coordination. The O—Cu—O angle is 80.07 (8)° and the P—Cu—P angle is 123.49 (3)°. The crystal packing is stablized by intra­molecular C—H⋯O inter­actions and inter­molecular C—H⋯O and O—H⋯O inter­actions.

## Related literature

The title compound is structurally related to the flavonolato, nitro­sophenyl­hydroxy­laminato and tropolonato derivatives, see: Spier *et al.* (1990 ▶); Charalambous *et al.* (1984 ▶); Steyl (2009 ▶). For related diketonato complexes, see: Odoko *et al.* (2002 ▶, 2003 ▶). For general background to pyran­one ligands, see: Hider *et al.* (1984**a* ▶,b* ▶); Kontoghiorghes *et al.* (1990 ▶); Kontoghiorghes (1995 ▶); Hedlund & Öhman (1988 ▶); Creeth *et al.* (2000 ▶). 
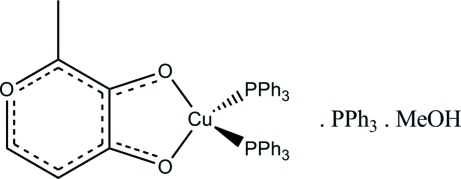


## Experimental

### 

#### Crystal data

[Cu(C_6_H_5_O_3_)(C_18_H_15_P)_2_]·C_18_H_15_P·CH_4_O
*M* *_r_* = 1007.49
Monoclinic, 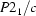

*a* = 20.5253 (7) Å
*b* = 13.5716 (4) Å
*c* = 20.3129 (7) Å
β = 119.205 (1)°
*V* = 4939.1 (3) Å^3^
*Z* = 4
Mo *K*α radiation
μ = 0.59 mm^−1^
*T* = 150 K
0.19 × 0.19 × 0.06 mm

#### Data collection

Bruker X8 APEXII 4K diffractometer
Absorption correction: multi-scan (*SADABS*; Bruker, 2004 ▶) *T*_min_ = 0.894, *T*_max_ = 0.965
58551 measured reflections
10787 independent reflections
8326 reflections with *I* > 2σ(*I*)
*R*_int_ = 0.058

#### Refinement

*R*[*F* ^2^ > 2σ(*F* ^2^)] = 0.049
*wR*(*F* ^2^) = 0.127
*S* = 1.06
10787 reflections
625 parameters
H-atom parameters constrained
Δρ_max_ = 1.51 e Å^−3^
Δρ_min_ = −0.86 e Å^−3^

### 

Data collection: *APEX2* (Bruker, 2005 ▶); cell refinement: *SAINT-Plus* (Bruker, 2004 ▶); data reduction: *SAINT-Plus*; program(s) used to solve structure: *SHELXS97* (Sheldrick, 2008 ▶); program(s) used to refine structure: *SHELXL97* (Sheldrick, 2008 ▶); molecular graphics: *DIAMOND* (Brandenburg & Putz, 2007 ▶); software used to prepare material for publication: *WinGX* (Farrugia, 1999 ▶).

## Supplementary Material

Crystal structure: contains datablocks global, I. DOI: 10.1107/S1600536811011470/fi2105sup1.cif

Structure factors: contains datablocks I. DOI: 10.1107/S1600536811011470/fi2105Isup2.hkl

Additional supplementary materials:  crystallographic information; 3D view; checkCIF report

## Figures and Tables

**Table 1:** Selected bond lengths (Å)

Cu1—O1	2.046 (2)
Cu1—O2	2.175 (2)
Cu1—P1	2.2014 (7)
Cu1—P2	2.2692 (8)

**Table 2:** Hydrogen-bond geometry (Å, °)

*D*—H⋯*A*	*D*—H	H⋯*A*	*D*⋯*A*	*D*—H⋯*A*
O4—H4*A*⋯O1	0.84	1.8	2.637 (3)	174
C2—H2⋯O2	0.95	2.46	3.370 (3)	162
C12—H12⋯O2	0.95	2.54	3.412 (4)	153
C22—H22⋯O4^i^	0.95	2.51	3.144 (4)	125
C32—H32⋯O1	0.95	2.6	3.495 (3)	158
C53—H53⋯O4	0.95	2.52	3.398 (5)	154

Symmetry code: (i) 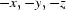.

**Fig. 1.**
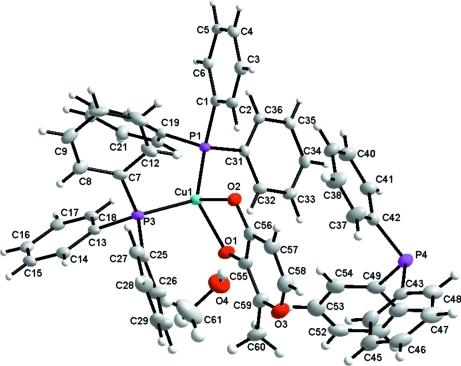
Molecular structure of the title compound. Thermal ellipsoids at 50% probability.

## supplementary crystallographic information

### Comment

Pyranone ligands have remarkable properties for clinical purposes (Odoko *et al.* 2003). These ligands are relevant to the control of metal levels in the body and have been tested for administration for the amelioration of anaemia (Hider *et al.* 1984**a*,b*) and the removal of iron (Kontoghiorghes *et al.* 1990) and aluminium (Kontoghiorghes, 1995). 3-hydroxy-2-methyl-4*H*- pyran-4-one is a naturally occurring non-toxic compound typically added as a food flavour enhancer. It has the ability to be deprotonated readily (pK_a_ = 8.38; Hedlund & Öhman, 1988) and can act as an anionic chelating *O,O'*-bidentate ligand towards a number of biologically active metal ions (Odoko *et al.* 2002). The efficacy of the Cu^II^ and Sn^II^ complexes in oral-care formations (Creeth *et al.* 2000) has also been reported. Only three other examples of copper triphenylphosphine complexes are known to date, which containes a five-membered *O,O'*-bidentate chelating ring system, *i.e*., the flavonolato, nitrosophenylhydroxylaminato and tropolonato derivatives (Spier *et al.*, 1990; Charalambous *et al.* 1984; Steyl, 2009). In this paper, the structure of (2-methyl-4-oxo-4*H*-pyran-3-olato- κ^2^*O*^3^,*O*^4^) Copper(I) complex is reported (Fig. 1). The pyran-4-one ring is essentially planar (r.m.s = 0.0138 fitted atoms C55, C56, C57, C58, C59 and O3). The Cu atom is situated 0.4508 (48) Å above the pyran-4-one ring plane. The Cu—O1 and Cu—O2 bond lengths are 2.046 (2) Å and 2.175 Å, respectively, this correlates well with literature (Steyl, 2009). The bidentate bite angle O1—Cu—O2 is 80.07 (8) ° which correlates with the observed literature values (Odoko *et al.* 2003). The Cu—P1 and Cu—P2 bond length is 2.2014 (7) and 2.2692 (8) Å, respectively, this is within normal range (Spier *et al.* 1990; Charalambous *et al.* 1984). The P1—Cu—P2 bond angle is 123.49 (3) °. O4–H4A···O1 hydrogen interactions between the solvent molecule and the complex and C53—H53···O1 hydrogen interaction between the free phosphie and complex stabilze the crystal packing. The crystal is further stabilized by inter- and intramolecular C—H···O hydrogen interactions (Table 2).

### Experimental

A solution of [Cu(NO_3_)(PPh_3_)_2_] (0.6502 g, 0.001 mol) in methanol (10 ml) was slowly added to a solution of 3-hydroxy-2-methyl-4*H*-pyran-4-one (0.1387 g, 0.0011 mol) in methanol (10 ml) and stirred for 30 minutes. Recrystallization from methanol gave X-Ray quality crystals. Yield 78%.

### Refinement

All H atoms were positioned geometrically and allowed to ride on their parent atoms, with *U*_iso_(H) = 1.2U_eq_(parent) of the parent atom with a C—H distance of 0.93.The methyl H atoms were placed in geometrically idealized positions and constrained to ride on its parent atoms with *U*_iso_(H) = 1.5*U*_eq_(C) and at a distance of 0.96 Å. The highest peak in the Fourier map (1.51 e.Å^–3^) is located 0.83Å from Cu1.

### Crystal data

**Table:**

[Cu(C_6_H_5_O_3_)(C_18_H_15_P)_2_]·C_18_H_15_P·CH_4_O	*F*(000) = 2104
*M**_r_* = 1007.49	*D*_x_ = 1.355 Mg m^−^^3^
Monoclinic, *P*2_1_/*c*	Mo *K*α radiation, λ = 0.71073 Å
Hall symbol: -P 2yc	Cell parameters from 8938 reflections
*a* = 20.5253 (7) Å	θ = 2.3–26.4°
*b* = 13.5716 (4) Å	µ = 0.59 mm^−^^1^
*c* = 20.3129 (7) Å	*T* = 150 K
β = 119.205 (1)°	Plate, colourless
*V* = 4939.1 (3) Å^3^	0.19 × 0.19 × 0.06 mm
*Z* = 4	

### Data collection

**Table:**

Bruker X8 APEXII 4K diffractometer	10787 independent reflections
Radiation source: fine-focus sealed tube	8326 reflections with *I* > 2σ(*I*)
graphite	*R*_int_ = 0.058
φ and ω scans	θ_max_ = 27°, θ_min_ = 1.9°
Absorption correction: multi-scan (*SADABS*; Bruker, 2004)	*h* = −26→26
*T*_min_ = 0.894, *T*_max_ = 0.965	*k* = −17→17
58551 measured reflections	*l* = −24→25

### Refinement

**Table:**

Refinement on *F*^2^	Primary atom site location: structure-invariant direct methods
Least-squares matrix: full	Secondary atom site location: difference Fourier map
*R*[*F*^2^ > 2σ(*F*^2^)] = 0.049	Hydrogen site location: inferred from neighbouring sites
*wR*(*F*^2^) = 0.127	H-atom parameters constrained
*S* = 1.06	*w* = 1/[σ^2^(*F*_o_^2^) + (0.0475*P*)^2^ + 7.9566*P*] where *P* = (*F*_o_^2^ + 2*F*_c_^2^)/3
10787 reflections	(Δ/σ)_max_ = 0.001
625 parameters	Δρ_max_ = 1.51 e Å^−^^3^
0 restraints	Δρ_min_ = −0.86 e Å^−^^3^

### Special details

**Table:**

Experimental. The intensity data was collected on a Bruker X8 ApexII 4 K Kappa CCD diffractometer using an exposure time of 60 s/frame. A total of 688 frames were collected with a frame width of 0.5° covering up to θ = 28.24° with 99.1% completeness accomplished.
Geometry. All s.u.'s (except the s.u. in the dihedral angle between two l.s. planes) are estimated using the full covariance matrix. The cell s.u.'s are taken into account individually in the estimation of s.u.'s in distances, angles and torsion angles; correlations between s.u.'s in cell parameters are only used when they are defined by crystal symmetry. An approximate (isotropic) treatment of cell s.u.'s is used for estimating s.u.'s involving l.s. planes.
Refinement. Refinement of *F*^2^ against ALL reflections. The weighted *R*-factor *wR* and goodness of fit *S* are based on *F*^2^, conventional *R*-factors *R* are based on *F*, with *F* set to zero for negative *F*^2^. The threshold expression of *F*^2^ > 2σ(*F*^2^) is used only for calculating *R*-factors(gt) *etc*. and is not relevant to the choice of reflections for refinement. *R*-factors based on *F*^2^ are statistically about twice as large as those based on *F*, and *R*- factors based on ALL data will be even larger.

### Fractional atomic coordinates and isotropic or equivalent isotropic displacement parameters (Å^2^)

**Table:**

	*x*	*y*	*z*	*U*_iso_*/*U*_eq_	
Cu1	0.214103 (18)	0.00326 (2)	0.261886 (19)	0.01908 (9)	
P1	0.10439 (4)	0.06950 (5)	0.22835 (4)	0.01542 (14)	
P2	0.23466 (4)	−0.16169 (5)	0.26969 (4)	0.01741 (15)	
P3	0.38902 (4)	0.48977 (6)	0.31013 (4)	0.02500 (17)	
O1	0.29079 (11)	0.06514 (16)	0.23758 (11)	0.0265 (5)	
O2	0.29997 (11)	0.04399 (15)	0.37487 (11)	0.0245 (4)	
C1	0.08207 (15)	0.08316 (18)	0.30441 (15)	0.0164 (5)	
C2	0.14009 (15)	0.08692 (19)	0.37862 (16)	0.0199 (6)	
H2	0.1903	0.0819	0.3888	0.024*	
C6	0.00836 (15)	0.09007 (18)	0.29104 (15)	0.0170 (5)	
H6	−0.0319	0.0861	0.2409	0.02*	
C5	−0.00614 (15)	0.10258 (19)	0.35027 (16)	0.0186 (5)	
H5	−0.0562	0.1091	0.3405	0.022*	
C3	0.12498 (16)	0.0979 (2)	0.43784 (16)	0.0217 (6)	
H3	0.1649	0.1001	0.4883	0.026*	
C4	0.05204 (16)	0.1056 (2)	0.42358 (16)	0.0210 (6)	
H4	0.0419	0.113	0.4642	0.025*	
C7	0.20878 (15)	−0.2199 (2)	0.33478 (16)	0.0202 (6)	
C8	0.18613 (16)	−0.3169 (2)	0.33070 (17)	0.0261 (6)	
H8	0.1824	−0.358	0.2911	0.031*	
C10	0.17415 (17)	−0.2963 (2)	0.44152 (18)	0.0298 (7)	
H10	0.1616	−0.3219	0.4774	0.036*	
C9	0.16883 (16)	−0.3550 (2)	0.38402 (17)	0.0291 (7)	
H9	0.1533	−0.4216	0.3806	0.035*	
C12	0.21461 (19)	−0.1614 (2)	0.39378 (19)	0.0316 (7)	
H12	0.2302	−0.0947	0.3976	0.038*	
C11	0.1978 (2)	−0.2001 (3)	0.4469 (2)	0.0361 (8)	
H11	0.2026	−0.16	0.4874	0.043*	
C14	0.22245 (15)	−0.3304 (2)	0.18224 (16)	0.0206 (6)	
H14	0.2683	−0.3519	0.2234	0.025*	
C13	0.19240 (15)	−0.2392 (2)	0.18613 (15)	0.0185 (5)	
C18	0.12553 (15)	−0.2083 (2)	0.12403 (16)	0.0193 (6)	
H18	0.1048	−0.1462	0.1254	0.023*	
C17	0.08922 (15)	−0.2673 (2)	0.06064 (16)	0.0215 (6)	
H17	0.044	−0.2455	0.0187	0.026*	
C15	0.18590 (16)	−0.3892 (2)	0.11902 (16)	0.0217 (6)	
H15	0.2067	−0.451	0.1171	0.026*	
C16	0.11908 (16)	−0.3586 (2)	0.05846 (16)	0.0216 (6)	
H16	0.0937	−0.3998	0.0155	0.026*	
C23	−0.05007 (16)	−0.1488 (2)	0.13305 (17)	0.0223 (6)	
H23	−0.0624	−0.2075	0.15	0.027*	
C19	0.02499 (14)	−0.00172 (19)	0.15983 (15)	0.0159 (5)	
C24	0.00684 (15)	−0.08956 (19)	0.18378 (16)	0.0196 (6)	
H24	0.034	−0.1083	0.2353	0.023*	
C20	−0.01466 (16)	0.0231 (2)	0.08419 (16)	0.0219 (6)	
H20	−0.0029	0.082	0.067	0.026*	
C22	−0.08934 (17)	−0.1231 (2)	0.05755 (18)	0.0265 (6)	
H22	−0.1284	−0.1643	0.0226	0.032*	
C21	−0.07135 (17)	−0.0373 (2)	0.03344 (17)	0.0262 (6)	
H21	−0.0981	−0.0195	−0.0183	0.031*	
C27	0.37303 (16)	−0.2392 (2)	0.37962 (17)	0.0264 (6)	
H27	0.3475	−0.2627	0.4049	0.032*	
C25	0.33395 (14)	−0.19082 (19)	0.31180 (15)	0.0183 (5)	
C26	0.37255 (17)	−0.1562 (2)	0.27614 (19)	0.0308 (7)	
H26	0.3466	−0.1217	0.2297	0.037*	
C28	0.44955 (17)	−0.2538 (3)	0.41136 (18)	0.0333 (7)	
H28	0.4759	−0.2875	0.4581	0.04*	
C29	0.48730 (17)	−0.2200 (2)	0.3756 (2)	0.0333 (7)	
H29	0.5396	−0.23	0.3974	0.04*	
C30	0.44870 (18)	−0.1718 (3)	0.3081 (2)	0.0364 (8)	
H30	0.4744	−0.1488	0.2829	0.044*	
C31	0.08839 (15)	0.19196 (18)	0.18591 (15)	0.0165 (5)	
C34	0.07028 (16)	0.3770 (2)	0.11999 (15)	0.0208 (6)	
H34	0.0645	0.4404	0.098	0.025*	
C35	0.02073 (15)	0.34515 (19)	0.14352 (15)	0.0187 (5)	
H35	−0.0192	0.3865	0.1374	0.022*	
C36	0.02951 (15)	0.25301 (19)	0.17593 (15)	0.0181 (5)	
H36	−0.0048	0.2311	0.1915	0.022*	
C32	0.13759 (15)	0.2245 (2)	0.16215 (16)	0.0211 (6)	
H32	0.178	0.1837	0.1689	0.025*	
C33	0.12819 (16)	0.3166 (2)	0.12855 (17)	0.0233 (6)	
H33	0.1615	0.3379	0.1115	0.028*	
C42	0.33225 (16)	0.4130 (2)	0.33699 (16)	0.0214 (6)	
C41	0.25678 (17)	0.4336 (2)	0.30419 (18)	0.0285 (7)	
H41	0.2355	0.4815	0.2652	0.034*	
C40	0.21165 (17)	0.3860 (2)	0.32715 (19)	0.0314 (7)	
H40	0.1598	0.4004	0.3033	0.038*	
C39	0.24148 (18)	0.3180 (2)	0.3842 (2)	0.0328 (7)	
H39	0.2107	0.2857	0.4004	0.039*	
C38	0.3168 (2)	0.2969 (3)	0.4180 (2)	0.0418 (9)	
H38	0.3381	0.2505	0.458	0.05*	
C37	0.36142 (19)	0.3430 (3)	0.3936 (2)	0.0378 (8)	
H37	0.4128	0.3263	0.4162	0.045*	
C44	0.50530 (17)	0.3496 (2)	0.34617 (19)	0.0327 (7)	
H44	0.472	0.3155	0.3014	0.039*	
C43	0.48189 (16)	0.4346 (2)	0.36526 (17)	0.0263 (6)	
C45	0.57669 (18)	0.3128 (3)	0.3913 (2)	0.0374 (8)	
H45	0.5924	0.2551	0.3765	0.045*	
C46	0.62451 (18)	0.3596 (3)	0.45730 (19)	0.0388 (8)	
H46	0.673	0.3337	0.4888	0.047*	
C47	0.60193 (19)	0.4443 (3)	0.4778 (2)	0.0451 (9)	
H47	0.6348	0.4766	0.5237	0.054*	
C48	0.53168 (18)	0.4823 (3)	0.43178 (19)	0.0371 (8)	
H48	0.5171	0.5417	0.4456	0.045*	
C49	0.36127 (16)	0.4483 (2)	0.21457 (17)	0.0283 (7)	
C54	0.33106 (17)	0.3582 (3)	0.18496 (18)	0.0318 (7)	
H54	0.3235	0.3112	0.2153	0.038*	
C51	0.35207 (18)	0.4942 (3)	0.0952 (2)	0.0396 (8)	
H51	0.3591	0.5409	0.0644	0.048*	
C50	0.37123 (17)	0.5176 (3)	0.16850 (19)	0.0337 (7)	
H50	0.3913	0.5807	0.1881	0.04*	
C52	0.32287 (18)	0.4032 (3)	0.06709 (19)	0.0362 (8)	
H52	0.3105	0.387	0.0168	0.043*	
C53	0.31113 (18)	0.3348 (3)	0.1100 (2)	0.0373 (8)	
H53	0.2898	0.2726	0.0893	0.045*	
C55	0.35473 (16)	0.0786 (2)	0.29884 (17)	0.0236 (6)	
C59	0.41971 (16)	0.0984 (2)	0.29795 (18)	0.0282 (7)	
C60	0.42869 (18)	0.1052 (3)	0.2299 (2)	0.0363 (8)	
H60A	0.4396	0.0398	0.2174	0.054*	
H60B	0.4699	0.1501	0.2399	0.054*	
H60C	0.3825	0.1303	0.1875	0.054*	
C57	0.42829 (17)	0.0856 (2)	0.43665 (19)	0.0304 (7)	
H57	0.4327	0.0833	0.4854	0.036*	
C58	0.48828 (18)	0.1051 (3)	0.42923 (19)	0.0348 (7)	
H58	0.5347	0.1156	0.4736	0.042*	
C56	0.35775 (16)	0.0683 (2)	0.37156 (17)	0.0236 (6)	
O3	0.48634 (12)	0.11061 (17)	0.36300 (13)	0.0350 (5)	
O4	0.24131 (15)	0.1036 (2)	0.09364 (14)	0.0463 (6)	
H4A	0.2582	0.0956	0.1402	0.069*	
C61	0.2624 (3)	0.0252 (4)	0.0650 (3)	0.0755 (16)	
H61A	0.3164	0.0274	0.0843	0.113*	
H61B	0.2365	0.0293	0.0098	0.113*	
H61C	0.2491	−0.0367	0.0804	0.113*	

### Atomic displacement parameters (Å^2^)

**Table:**

	*U*^11^	*U*^22^	*U*^33^	*U*^12^	*U*^13^	*U*^23^
Cu1	0.01644 (17)	0.01898 (17)	0.02494 (18)	0.00279 (13)	0.01253 (14)	0.00323 (14)
P1	0.0157 (3)	0.0140 (3)	0.0198 (3)	0.0010 (2)	0.0112 (3)	0.0015 (3)
P2	0.0167 (3)	0.0182 (3)	0.0201 (3)	0.0038 (3)	0.0112 (3)	0.0026 (3)
P3	0.0204 (4)	0.0275 (4)	0.0260 (4)	0.0003 (3)	0.0105 (3)	0.0044 (3)
O1	0.0213 (10)	0.0334 (12)	0.0270 (11)	−0.0004 (9)	0.0134 (9)	0.0037 (9)
O2	0.0221 (10)	0.0272 (11)	0.0258 (11)	−0.0037 (8)	0.0129 (9)	−0.0025 (9)
C1	0.0201 (13)	0.0111 (12)	0.0216 (13)	0.0005 (10)	0.0131 (11)	0.0012 (10)
C2	0.0180 (13)	0.0181 (13)	0.0265 (15)	0.0014 (10)	0.0131 (12)	0.0004 (11)
C6	0.0196 (13)	0.0133 (12)	0.0194 (13)	0.0004 (10)	0.0105 (11)	0.0008 (10)
C5	0.0191 (13)	0.0151 (12)	0.0276 (15)	0.0004 (10)	0.0161 (12)	0.0009 (11)
C3	0.0229 (14)	0.0207 (14)	0.0206 (14)	0.0004 (11)	0.0097 (12)	−0.0002 (11)
C4	0.0278 (15)	0.0182 (13)	0.0218 (14)	−0.0001 (11)	0.0156 (12)	0.0000 (11)
C7	0.0176 (13)	0.0250 (14)	0.0218 (14)	0.0058 (11)	0.0127 (12)	0.0042 (11)
C8	0.0233 (15)	0.0327 (16)	0.0210 (14)	−0.0047 (12)	0.0097 (12)	0.0004 (12)
C10	0.0239 (15)	0.0439 (19)	0.0311 (16)	0.0084 (13)	0.0209 (14)	0.0124 (14)
C9	0.0211 (15)	0.0374 (17)	0.0240 (15)	−0.0066 (13)	0.0073 (13)	0.0074 (13)
C12	0.046 (2)	0.0251 (16)	0.0373 (18)	0.0088 (14)	0.0313 (16)	0.0043 (13)
C11	0.053 (2)	0.0339 (18)	0.0385 (19)	0.0141 (16)	0.0352 (18)	0.0070 (15)
C14	0.0190 (13)	0.0220 (14)	0.0227 (14)	0.0033 (11)	0.0117 (12)	0.0042 (11)
C13	0.0207 (13)	0.0191 (13)	0.0219 (14)	0.0013 (11)	0.0152 (12)	0.0027 (11)
C18	0.0209 (14)	0.0193 (13)	0.0239 (14)	0.0045 (11)	0.0156 (12)	0.0040 (11)
C17	0.0194 (14)	0.0277 (15)	0.0202 (14)	0.0020 (11)	0.0118 (12)	0.0076 (12)
C15	0.0239 (14)	0.0205 (14)	0.0273 (15)	0.0035 (11)	0.0176 (13)	0.0024 (11)
C16	0.0247 (14)	0.0251 (14)	0.0215 (14)	−0.0044 (11)	0.0164 (12)	−0.0002 (11)
C23	0.0267 (15)	0.0149 (13)	0.0330 (16)	0.0005 (11)	0.0206 (13)	−0.0002 (11)
C19	0.0163 (12)	0.0144 (12)	0.0211 (13)	0.0026 (10)	0.0122 (11)	0.0004 (10)
C24	0.0189 (13)	0.0165 (13)	0.0262 (15)	0.0038 (10)	0.0133 (12)	0.0036 (11)
C20	0.0268 (15)	0.0184 (14)	0.0265 (15)	−0.0004 (11)	0.0177 (13)	0.0006 (11)
C22	0.0266 (15)	0.0243 (15)	0.0323 (16)	−0.0077 (12)	0.0172 (14)	−0.0091 (13)
C21	0.0278 (16)	0.0285 (15)	0.0227 (15)	−0.0039 (12)	0.0125 (13)	−0.0027 (12)
C27	0.0226 (15)	0.0313 (16)	0.0244 (15)	0.0017 (12)	0.0108 (13)	0.0001 (12)
C25	0.0151 (13)	0.0179 (13)	0.0214 (14)	0.0022 (10)	0.0084 (11)	−0.0032 (11)
C26	0.0248 (16)	0.0393 (18)	0.0341 (17)	0.0093 (13)	0.0188 (14)	0.0096 (14)
C28	0.0227 (16)	0.0403 (19)	0.0270 (17)	0.0069 (13)	0.0045 (13)	0.0024 (14)
C29	0.0170 (14)	0.0339 (17)	0.047 (2)	−0.0009 (13)	0.0140 (15)	−0.0096 (15)
C30	0.0271 (17)	0.0401 (19)	0.052 (2)	0.0040 (14)	0.0270 (17)	0.0041 (16)
C31	0.0200 (13)	0.0132 (12)	0.0176 (13)	−0.0001 (10)	0.0102 (11)	−0.0004 (10)
C34	0.0287 (15)	0.0138 (13)	0.0209 (14)	−0.0004 (11)	0.0129 (12)	0.0026 (11)
C35	0.0228 (14)	0.0158 (13)	0.0195 (13)	0.0020 (10)	0.0119 (12)	−0.0012 (10)
C36	0.0171 (13)	0.0188 (13)	0.0200 (13)	−0.0031 (10)	0.0104 (11)	−0.0008 (10)
C32	0.0213 (14)	0.0207 (14)	0.0258 (15)	0.0030 (11)	0.0149 (12)	0.0023 (11)
C33	0.0256 (15)	0.0234 (14)	0.0279 (15)	0.0000 (11)	0.0187 (13)	0.0041 (12)
C42	0.0208 (14)	0.0193 (13)	0.0249 (15)	0.0013 (11)	0.0118 (12)	−0.0015 (11)
C41	0.0269 (16)	0.0296 (16)	0.0320 (17)	0.0054 (12)	0.0168 (14)	0.0059 (13)
C40	0.0204 (15)	0.0381 (18)	0.0352 (18)	0.0008 (13)	0.0132 (14)	0.0007 (14)
C39	0.0324 (17)	0.0281 (16)	0.047 (2)	−0.0045 (13)	0.0262 (16)	0.0013 (14)
C38	0.039 (2)	0.041 (2)	0.052 (2)	0.0076 (16)	0.0277 (18)	0.0205 (17)
C37	0.0256 (17)	0.0418 (19)	0.046 (2)	0.0063 (14)	0.0171 (16)	0.0167 (16)
C44	0.0226 (15)	0.0343 (17)	0.0345 (18)	−0.0013 (13)	0.0087 (14)	0.0051 (14)
C43	0.0192 (14)	0.0346 (17)	0.0265 (15)	−0.0016 (12)	0.0123 (13)	0.0073 (13)
C45	0.0253 (17)	0.0384 (19)	0.048 (2)	0.0052 (14)	0.0170 (16)	0.0120 (16)
C46	0.0211 (16)	0.063 (2)	0.0319 (18)	0.0039 (15)	0.0125 (14)	0.0146 (17)
C47	0.0267 (18)	0.077 (3)	0.0277 (18)	−0.0047 (18)	0.0105 (15)	−0.0073 (18)
C48	0.0290 (17)	0.055 (2)	0.0305 (17)	−0.0007 (15)	0.0173 (15)	−0.0044 (16)
C49	0.0170 (14)	0.0427 (18)	0.0238 (15)	0.0050 (13)	0.0089 (12)	0.0031 (13)
C54	0.0213 (15)	0.0408 (19)	0.0314 (17)	0.0077 (13)	0.0112 (14)	0.0040 (14)
C51	0.0275 (17)	0.059 (2)	0.0343 (18)	0.0030 (16)	0.0166 (15)	0.0109 (17)
C50	0.0218 (15)	0.045 (2)	0.0343 (18)	0.0018 (14)	0.0134 (14)	0.0056 (15)
C52	0.0234 (16)	0.059 (2)	0.0291 (17)	0.0069 (15)	0.0150 (14)	−0.0015 (16)
C53	0.0230 (16)	0.043 (2)	0.0386 (19)	0.0034 (14)	0.0091 (15)	−0.0041 (16)
C55	0.0186 (14)	0.0236 (14)	0.0289 (16)	0.0030 (11)	0.0119 (13)	0.0042 (12)
C59	0.0202 (15)	0.0299 (16)	0.0331 (17)	0.0007 (12)	0.0118 (13)	0.0032 (13)
C60	0.0259 (16)	0.045 (2)	0.046 (2)	0.0012 (14)	0.0237 (16)	0.0066 (16)
C57	0.0250 (16)	0.0336 (17)	0.0290 (17)	0.0007 (13)	0.0103 (14)	−0.0004 (13)
C58	0.0262 (16)	0.0382 (18)	0.0331 (18)	−0.0009 (14)	0.0090 (14)	−0.0012 (15)
C56	0.0227 (15)	0.0191 (14)	0.0294 (16)	0.0008 (11)	0.0130 (13)	0.0001 (12)
O3	0.0185 (11)	0.0428 (13)	0.0398 (13)	−0.0019 (9)	0.0111 (10)	0.0018 (11)
O4	0.0463 (16)	0.0579 (17)	0.0320 (13)	0.0056 (13)	0.0170 (13)	−0.0006 (12)
C61	0.064 (3)	0.093 (4)	0.052 (3)	0.024 (3)	0.015 (2)	−0.028 (3)

### Geometric parameters (Å, °)

**Table:**

Cu1—O1	2.046 (2)	C28—H28	0.95
Cu1—O2	2.175 (2)	C29—C30	1.370 (5)
Cu1—P1	2.2014 (7)	C29—H29	0.95
Cu1—P2	2.2692 (8)	C30—H30	0.95
P1—C19	1.820 (3)	C31—C32	1.387 (4)
P1—C1	1.823 (3)	C31—C36	1.396 (4)
P1—C31	1.827 (3)	C34—C33	1.384 (4)
P2—C13	1.817 (3)	C34—C35	1.387 (4)
P2—C7	1.827 (3)	C34—H34	0.95
P2—C25	1.828 (3)	C35—C36	1.383 (4)
P3—C49	1.825 (3)	C35—H35	0.95
P3—C42	1.833 (3)	C36—H36	0.95
P3—C43	1.834 (3)	C32—C33	1.391 (4)
O1—C55	1.308 (4)	C32—H32	0.95
O2—C56	1.264 (3)	C33—H33	0.95
C1—C2	1.393 (4)	C42—C37	1.383 (4)
C1—C6	1.404 (4)	C42—C41	1.384 (4)
C2—C3	1.389 (4)	C41—C40	1.384 (4)
C2—H2	0.95	C41—H41	0.95
C6—C5	1.384 (4)	C40—C39	1.370 (5)
C6—H6	0.95	C40—H40	0.95
C5—C4	1.382 (4)	C39—C38	1.382 (5)
C5—H5	0.95	C39—H39	0.95
C3—C4	1.383 (4)	C38—C37	1.384 (5)
C3—H3	0.95	C38—H38	0.95
C4—H4	0.95	C37—H37	0.95
C7—C8	1.386 (4)	C44—C43	1.377 (5)
C7—C12	1.393 (4)	C44—C45	1.388 (4)
C8—C9	1.393 (4)	C44—H44	0.95
C8—H8	0.95	C43—C48	1.393 (5)
C10—C9	1.373 (5)	C45—C46	1.372 (5)
C10—C11	1.379 (5)	C45—H45	0.95
C10—H10	0.95	C46—C47	1.378 (5)
C9—H9	0.95	C46—H46	0.95
C12—C11	1.387 (4)	C47—C48	1.379 (5)
C12—H12	0.95	C47—H47	0.95
C11—H11	0.95	C48—H48	0.95
C14—C15	1.382 (4)	C49—C54	1.370 (5)
C14—C13	1.402 (4)	C49—C50	1.410 (5)
C14—H14	0.95	C54—C53	1.407 (5)
C13—C18	1.400 (4)	C54—H54	0.95
C18—C17	1.385 (4)	C51—C52	1.371 (5)
C18—H18	0.95	C51—C50	1.379 (5)
C17—C16	1.392 (4)	C51—H51	0.95
C17—H17	0.95	C50—H50	0.95
C15—C16	1.386 (4)	C52—C53	1.372 (5)
C15—H15	0.95	C52—H52	0.95
C16—H16	0.95	C53—H53	0.95
C23—C24	1.378 (4)	C55—C59	1.369 (4)
C23—C22	1.384 (4)	C55—C56	1.455 (4)
C23—H23	0.95	C59—O3	1.371 (4)
C19—C20	1.384 (4)	C59—C60	1.483 (5)
C19—C24	1.405 (4)	C60—H60A	0.98
C24—H24	0.95	C60—H60B	0.98
C20—C21	1.384 (4)	C60—H60C	0.98
C20—H20	0.95	C57—C58	1.338 (5)
C22—C21	1.383 (4)	C57—C56	1.425 (4)
C22—H22	0.95	C57—H57	0.95
C21—H21	0.95	C58—O3	1.328 (4)
C27—C25	1.376 (4)	C58—H58	0.95
C27—C28	1.391 (4)	O4—C61	1.379 (5)
C27—H27	0.95	O4—H4A	0.84
C25—C26	1.390 (4)	C61—H61A	0.98
C26—C30	1.386 (4)	C61—H61B	0.98
C26—H26	0.95	C61—H61C	0.98
C28—C29	1.374 (5)		
			
O1—Cu1—O2	80.07 (8)	C30—C29—C28	119.3 (3)
O1—Cu1—P1	123.30 (6)	C30—C29—H29	120.4
O2—Cu1—P1	113.74 (6)	C28—C29—H29	120.4
O1—Cu1—P2	106.44 (6)	C29—C30—C26	120.7 (3)
O2—Cu1—P2	98.61 (6)	C29—C30—H30	119.6
P1—Cu1—P2	123.49 (3)	C26—C30—H30	119.6
C19—P1—C1	101.57 (12)	C32—C31—C36	119.1 (2)
C19—P1—C31	103.56 (12)	C32—C31—P1	117.4 (2)
C1—P1—C31	104.23 (12)	C36—C31—P1	123.5 (2)
C19—P1—Cu1	114.75 (8)	C33—C34—C35	120.1 (2)
C1—P1—Cu1	114.93 (9)	C33—C34—H34	120
C31—P1—Cu1	116.03 (9)	C35—C34—H34	120
C13—P2—C7	104.53 (13)	C36—C35—C34	119.9 (3)
C13—P2—C25	103.13 (12)	C36—C35—H35	120
C7—P2—C25	102.67 (12)	C34—C35—H35	120
C13—P2—Cu1	121.19 (9)	C35—C36—C31	120.5 (2)
C7—P2—Cu1	111.53 (9)	C35—C36—H36	119.7
C25—P2—Cu1	111.90 (9)	C31—C36—H36	119.7
C49—P3—C42	103.15 (14)	C31—C32—C33	120.4 (3)
C49—P3—C43	102.45 (14)	C31—C32—H32	119.8
C42—P3—C43	102.04 (13)	C33—C32—H32	119.8
C55—O1—Cu1	111.17 (18)	C34—C33—C32	120.0 (3)
C56—O2—Cu1	107.97 (18)	C34—C33—H33	120
C2—C1—C6	118.6 (2)	C32—C33—H33	120
C2—C1—P1	119.0 (2)	C37—C42—C41	117.9 (3)
C6—C1—P1	122.4 (2)	C37—C42—P3	123.9 (2)
C3—C2—C1	120.4 (3)	C41—C42—P3	117.8 (2)
C3—C2—H2	119.8	C42—C41—C40	121.3 (3)
C1—C2—H2	119.8	C42—C41—H41	119.4
C5—C6—C1	120.6 (3)	C40—C41—H41	119.4
C5—C6—H6	119.7	C39—C40—C41	120.2 (3)
C1—C6—H6	119.7	C39—C40—H40	119.9
C4—C5—C6	120.1 (3)	C41—C40—H40	119.9
C4—C5—H5	120	C40—C39—C38	119.3 (3)
C6—C5—H5	120	C40—C39—H39	120.3
C4—C3—C2	120.2 (3)	C38—C39—H39	120.3
C4—C3—H3	119.9	C39—C38—C37	120.3 (3)
C2—C3—H3	119.9	C39—C38—H38	119.9
C5—C4—C3	120.1 (3)	C37—C38—H38	119.9
C5—C4—H4	120	C42—C37—C38	121.0 (3)
C3—C4—H4	120	C42—C37—H37	119.5
C8—C7—C12	118.6 (3)	C38—C37—H37	119.5
C8—C7—P2	125.1 (2)	C43—C44—C45	121.1 (3)
C12—C7—P2	116.3 (2)	C43—C44—H44	119.4
C7—C8—C9	120.7 (3)	C45—C44—H44	119.4
C7—C8—H8	119.6	C44—C43—C48	118.1 (3)
C9—C8—H8	119.6	C44—C43—P3	124.9 (2)
C9—C10—C11	119.7 (3)	C48—C43—P3	117.1 (2)
C9—C10—H10	120.1	C46—C45—C44	120.0 (3)
C11—C10—H10	120.1	C46—C45—H45	120
C10—C9—C8	120.1 (3)	C44—C45—H45	120
C10—C9—H9	119.9	C45—C46—C47	119.8 (3)
C8—C9—H9	119.9	C45—C46—H46	120.1
C11—C12—C7	120.3 (3)	C47—C46—H46	120.1
C11—C12—H12	119.9	C46—C47—C48	120.0 (3)
C7—C12—H12	119.9	C46—C47—H47	120
C10—C11—C12	120.6 (3)	C48—C47—H47	120
C10—C11—H11	119.7	C47—C48—C43	120.9 (3)
C12—C11—H11	119.7	C47—C48—H48	119.5
C15—C14—C13	120.5 (3)	C43—C48—H48	119.5
C15—C14—H14	119.7	C54—C49—C50	119.0 (3)
C13—C14—H14	119.7	C54—C49—P3	125.6 (3)
C18—C13—C14	118.5 (3)	C50—C49—P3	115.4 (3)
C18—C13—P2	118.7 (2)	C49—C54—C53	120.4 (3)
C14—C13—P2	122.7 (2)	C49—C54—H54	119.8
C17—C18—C13	120.7 (3)	C53—C54—H54	119.8
C17—C18—H18	119.6	C52—C51—C50	119.5 (3)
C13—C18—H18	119.6	C52—C51—H51	120.2
C18—C17—C16	119.9 (3)	C50—C51—H51	120.2
C18—C17—H17	120.1	C51—C50—C49	120.4 (3)
C16—C17—H17	120.1	C51—C50—H50	119.8
C14—C15—C16	120.3 (3)	C49—C50—H50	119.8
C14—C15—H15	119.8	C51—C52—C53	121.4 (3)
C16—C15—H15	119.8	C51—C52—H52	119.3
C15—C16—C17	119.9 (3)	C53—C52—H52	119.3
C15—C16—H16	120	C52—C53—C54	119.1 (3)
C17—C16—H16	120	C52—C53—H53	120.4
C24—C23—C22	120.3 (3)	C54—C53—H53	120.4
C24—C23—H23	119.8	O1—C55—C59	123.1 (3)
C22—C23—H23	119.8	O1—C55—C56	118.6 (2)
C20—C19—C24	118.6 (3)	C59—C55—C56	118.2 (3)
C20—C19—P1	122.8 (2)	C55—C59—O3	122.1 (3)
C24—C19—P1	118.4 (2)	C55—C59—C60	126.1 (3)
C23—C24—C19	120.5 (3)	O3—C59—C60	111.8 (3)
C23—C24—H24	119.8	C59—C60—H60A	109.5
C19—C24—H24	119.8	C59—C60—H60B	109.5
C19—C20—C21	120.5 (3)	H60A—C60—H60B	109.5
C19—C20—H20	119.7	C59—C60—H60C	109.5
C21—C20—H20	119.7	H60A—C60—H60C	109.5
C21—C22—C23	119.5 (3)	H60B—C60—H60C	109.5
C21—C22—H22	120.2	C58—C57—C56	120.2 (3)
C23—C22—H22	120.2	C58—C57—H57	119.9
C22—C21—C20	120.5 (3)	C56—C57—H57	119.9
C22—C21—H21	119.7	O3—C58—C57	123.4 (3)
C20—C21—H21	119.7	O3—C58—H58	118.3
C25—C27—C28	120.5 (3)	C57—C58—H58	118.3
C25—C27—H27	119.8	O2—C56—C57	123.3 (3)
C28—C27—H27	119.8	O2—C56—C55	120.2 (3)
C27—C25—C26	118.7 (3)	C57—C56—C55	116.5 (3)
C27—C25—P2	123.0 (2)	C58—O3—C59	119.5 (3)
C26—C25—P2	118.2 (2)	C61—O4—H4A	109.5
C30—C26—C25	120.3 (3)	O4—C61—H61A	109.5
C30—C26—H26	119.9	O4—C61—H61B	109.5
C25—C26—H26	119.9	H61A—C61—H61B	109.5
C29—C28—C27	120.5 (3)	O4—C61—H61C	109.5
C29—C28—H28	119.7	H61A—C61—H61C	109.5
C27—C28—H28	119.7	H61B—C61—H61C	109.5
			
O1—Cu1—P1—C19	114.17 (12)	C28—C27—C25—P2	−175.9 (2)
O2—Cu1—P1—C19	−152.19 (11)	C13—P2—C25—C27	−112.7 (3)
P2—Cu1—P1—C19	−33.04 (10)	C7—P2—C25—C27	−4.2 (3)
O1—Cu1—P1—C1	−128.58 (12)	Cu1—P2—C25—C27	115.5 (2)
O2—Cu1—P1—C1	−34.94 (11)	C13—P2—C25—C26	72.0 (3)
P2—Cu1—P1—C1	84.21 (10)	C7—P2—C25—C26	−179.5 (2)
O1—Cu1—P1—C31	−6.66 (13)	Cu1—P2—C25—C26	−59.8 (2)
O2—Cu1—P1—C31	86.98 (11)	C27—C25—C26—C30	1.0 (5)
P2—Cu1—P1—C31	−153.88 (10)	P2—C25—C26—C30	176.5 (3)
O1—Cu1—P2—C13	−85.70 (12)	C25—C27—C28—C29	0.2 (5)
O2—Cu1—P2—C13	−167.81 (11)	C27—C28—C29—C30	−0.2 (5)
P1—Cu1—P2—C13	66.15 (11)	C28—C29—C30—C26	0.6 (5)
O1—Cu1—P2—C7	150.65 (11)	C25—C26—C30—C29	−1.0 (5)
O2—Cu1—P2—C7	68.54 (11)	C19—P1—C31—C32	−112.3 (2)
P1—Cu1—P2—C7	−57.51 (10)	C1—P1—C31—C32	141.8 (2)
O1—Cu1—P2—C25	36.28 (12)	Cu1—P1—C31—C32	14.4 (2)
O2—Cu1—P2—C25	−45.84 (11)	C19—P1—C31—C36	67.2 (2)
P1—Cu1—P2—C25	−171.88 (10)	C1—P1—C31—C36	−38.7 (3)
O2—Cu1—O1—C55	12.32 (19)	Cu1—P1—C31—C36	−166.1 (2)
P1—Cu1—O1—C55	124.29 (17)	C33—C34—C35—C36	0.3 (4)
P2—Cu1—O1—C55	−83.80 (19)	C34—C35—C36—C31	0.6 (4)
O1—Cu1—O2—C56	−11.20 (18)	C32—C31—C36—C35	−0.7 (4)
P1—Cu1—O2—C56	−133.33 (17)	P1—C31—C36—C35	179.8 (2)
P2—Cu1—O2—C56	94.12 (18)	C36—C31—C32—C33	−0.3 (4)
C19—P1—C1—C2	147.6 (2)	P1—C31—C32—C33	179.2 (2)
C31—P1—C1—C2	−105.0 (2)	C35—C34—C33—C32	−1.3 (4)
Cu1—P1—C1—C2	23.1 (2)	C31—C32—C33—C34	1.3 (4)
C19—P1—C1—C6	−32.4 (2)	C49—P3—C42—C37	114.1 (3)
C31—P1—C1—C6	75.0 (2)	C43—P3—C42—C37	8.1 (3)
Cu1—P1—C1—C6	−156.94 (18)	C49—P3—C42—C41	−73.1 (3)
C6—C1—C2—C3	−0.4 (4)	C43—P3—C42—C41	−179.1 (2)
P1—C1—C2—C3	179.6 (2)	C37—C42—C41—C40	0.0 (5)
C2—C1—C6—C5	1.4 (4)	P3—C42—C41—C40	−173.2 (3)
P1—C1—C6—C5	−178.6 (2)	C42—C41—C40—C39	1.1 (5)
C1—C6—C5—C4	−1.8 (4)	C41—C40—C39—C38	−0.6 (5)
C1—C2—C3—C4	−0.3 (4)	C40—C39—C38—C37	−0.9 (6)
C6—C5—C4—C3	1.1 (4)	C41—C42—C37—C38	−1.5 (5)
C2—C3—C4—C5	−0.1 (4)	P3—C42—C37—C38	171.3 (3)
C13—P2—C7—C8	20.2 (3)	C39—C38—C37—C42	2.0 (6)
C25—P2—C7—C8	−87.2 (3)	C45—C44—C43—C48	−0.8 (5)
Cu1—P2—C7—C8	152.8 (2)	C45—C44—C43—P3	−179.9 (2)
C13—P2—C7—C12	−161.6 (2)	C49—P3—C43—C44	−27.5 (3)
C25—P2—C7—C12	91.0 (2)	C42—P3—C43—C44	79.1 (3)
Cu1—P2—C7—C12	−29.0 (2)	C49—P3—C43—C48	153.4 (3)
C12—C7—C8—C9	0.6 (4)	C42—P3—C43—C48	−100.1 (3)
P2—C7—C8—C9	178.8 (2)	C43—C44—C45—C46	2.0 (5)
C11—C10—C9—C8	−1.0 (5)	C44—C45—C46—C47	−1.3 (5)
C7—C8—C9—C10	−0.1 (4)	C45—C46—C47—C48	−0.5 (5)
C8—C7—C12—C11	−0.2 (5)	C46—C47—C48—C43	1.8 (6)
P2—C7—C12—C11	−178.5 (3)	C44—C43—C48—C47	−1.1 (5)
C9—C10—C11—C12	1.5 (5)	P3—C43—C48—C47	178.1 (3)
C7—C12—C11—C10	−0.9 (5)	C42—P3—C49—C54	−23.6 (3)
C15—C14—C13—C18	−1.3 (4)	C43—P3—C49—C54	82.1 (3)
C15—C14—C13—P2	176.2 (2)	C42—P3—C49—C50	155.1 (2)
C7—P2—C13—C18	100.7 (2)	C43—P3—C49—C50	−99.2 (2)
C25—P2—C13—C18	−152.3 (2)	C50—C49—C54—C53	0.8 (4)
Cu1—P2—C13—C18	−26.2 (2)	P3—C49—C54—C53	179.4 (2)
C7—P2—C13—C14	−76.8 (2)	C52—C51—C50—C49	0.3 (5)
C25—P2—C13—C14	30.2 (3)	C54—C49—C50—C51	−1.2 (5)
Cu1—P2—C13—C14	156.28 (19)	P3—C49—C50—C51	−179.9 (2)
C14—C13—C18—C17	1.0 (4)	C50—C51—C52—C53	1.0 (5)
P2—C13—C18—C17	−176.6 (2)	C51—C52—C53—C54	−1.4 (5)
C13—C18—C17—C16	0.3 (4)	C49—C54—C53—C52	0.5 (5)
C13—C14—C15—C16	0.2 (4)	Cu1—O1—C55—C59	165.3 (2)
C14—C15—C16—C17	1.2 (4)	Cu1—O1—C55—C56	−12.1 (3)
C18—C17—C16—C15	−1.4 (4)	O1—C55—C59—O3	−178.9 (3)
C1—P1—C19—C20	132.2 (2)	C56—C55—C59—O3	−1.4 (4)
C31—P1—C19—C20	24.3 (3)	O1—C55—C59—C60	−1.5 (5)
Cu1—P1—C19—C20	−103.2 (2)	C56—C55—C59—C60	175.9 (3)
C1—P1—C19—C24	−52.8 (2)	C56—C57—C58—O3	0.7 (5)
C31—P1—C19—C24	−160.7 (2)	Cu1—O2—C56—C57	−170.7 (2)
Cu1—P1—C19—C24	71.9 (2)	Cu1—O2—C56—C55	8.5 (3)
C22—C23—C24—C19	1.0 (4)	C58—C57—C56—O2	175.8 (3)
C20—C19—C24—C23	−1.0 (4)	C58—C57—C56—C55	−3.4 (4)
P1—C19—C24—C23	−176.3 (2)	O1—C55—C56—O2	2.0 (4)
C24—C19—C20—C21	0.5 (4)	C59—C55—C56—O2	−175.6 (3)
P1—C19—C20—C21	175.5 (2)	O1—C55—C56—C57	−178.8 (3)
C24—C23—C22—C21	−0.4 (4)	C59—C55—C56—C57	3.7 (4)
C23—C22—C21—C20	−0.2 (4)	C57—C58—O3—C59	1.8 (5)
C19—C20—C21—C22	0.1 (4)	C55—C59—O3—C58	−1.3 (4)
C28—C27—C25—C26	−0.6 (4)	C60—C59—O3—C58	−179.0 (3)

### Hydrogen-bond geometry (Å, °)

**Table:**

*D*—H···*A*	*D*—H	H···*A*	*D*···*A*	*D*—H···*A*
O4—H4A···O1	0.84	1.8	2.637 (3)	174
C2—H2···O2	0.95	2.46	3.370 (3)	162
C12—H12···O2	0.95	2.54	3.412 (4)	153
C22—H22···O4^i^	0.95	2.51	3.144 (4)	125
C32—H32···O1	0.95	2.6	3.495 (3)	158
C53—H53···O4	0.95	2.52	3.398 (5)	154

Symmetry codes: (i) −*x*, −*y*, −*z*.
